# Role of Ionizing Radiation Techniques in Polymeric Hydrogel Synthesis for Tissue Engineering Applications

**DOI:** 10.3390/gels11010047

**Published:** 2025-01-08

**Authors:** Ion Călina, Maria Demeter, Anca Scărișoreanu, Awn Abbas, Muhammad Asim Raza

**Affiliations:** 1 National Institute for Laser, Plasma and Radiation Physics, 409 Atomiștilor, 077125 Măgurele, Romania; calina.cosmin@inflpr.ro (I.C.); maria.demeter@inflpr.ro (M.D.); anca.scarisoreanu@inflpr.ro (A.S.); 2 Department of Pharmacy, College of Veterinary Medicine, Sichuan Agricultural University, Chengdu 611130, China; 3 School of Chemical Engineering, Yeungnam University, Gyeongsan 38541, Republic of Korea

**Keywords:** ionizing radiation, hydrogels, crosslinking, polymerization, sterilization, tissue engineering

## Abstract

Hydrogels are widely utilized in industrial and scientific applications owing to their ability to immobilize active molecules, cells, and nanoparticles. This capability has led to their growing use in various biomedical fields, including cell culture and transplantation, drug delivery, and tissue engineering. Among the available synthesis techniques, ionizing-radiation-induced fabrication stands out as an environmentally friendly method for hydrogel preparation. In alignment with the current requirements for cleaner technologies, developing hydrogels using gamma and electron beam irradiation technologies represents a promising and innovative approach for their biomedical applications. A key advantage of these methods is their ability to synthesize homogeneous three-dimensional networks in a single step, without the need for chemical initiators or catalysts. Additionally, the fabrication process is controllable by adjusting the radiation dose and dose rate.

## 1. Introduction

Hydrogels are crosslinked hydrophilic polymer networks capable of absorbing water or biological fluids without degradation, making them a promising scaffolding substrate for three-dimensional (3D) cell culture in tissue engineering (TE). The effectiveness of hydrogels stems from their biocompatibility and resemblance to the extracellular matrix [1]. Crosslinking in hydrogels typically occurs through three mechanisms: physical crosslinking, chemical crosslinking, and ionizing-radiation-induced crosslinking. While physically crosslinked hydrogels exhibit insufficient mechanical strength, chemically crosslinked hydrogels require toxic initiators or crosslinkers [2]. Conversely, ionizing radiation technology offers a straightforward, efficient, and ambient-condition approach to hydrogel fabrication [3]. Within this framework, electron beam (E-beam) and gamma (γ) irradiation are the most commonly utilized methods [4].

Ionizing radiation has emerged as a preferred alternative to chemical crosslinking in biomedical applications as it ensures the chemical purity of the resulting hydrogels. To prepare hydrogels, aqueous solutions of synthetic hydrophilic polymers are typically crosslinked using ionizing radiation without requiring additional additives [5]. This method also enables the production of hydrogels with uniform nano- and macroscopic sizes owing to the high penetration capability of γ-radiation [6]. However, the production of hydrogels from natural polymers, such as polysaccharides, using this technology can result in the scission of polymeric chains, leading to a reduction in molecular weight without forming a gel [7,8]. To address this limitation, crosslinking blends of natural and synthetic polymers under ionizing radiation exposure has been proposed as an effective alternative [9,10,11,12].

Ionizing radiation can alter the molecular structure and macroscopic properties of polymeric materials [13]. In literature, various studies revealed that mechanical properties such as compression, tensile, flexural strength/modulus, and impact were improved up to some extent at the optimal radiation dose [14]. As per irradiation conditions, the mechanisms reveal two opposing trends: oxidative degradation, which often results in material weakening, and crosslinking of the polymer molecules, which enhances mechanical strength. It appears that the amount of oxygen present on the material and its capacity to fill up it when it is used up by chemical interactions with radicals created during irradiation will determine which phenomenon will predominate [15]. Furthermore, ionizing radiation can be used for materials of various states (i.e., liquid, solid, and gas) and at different temperatures [16,17,18]. Water-soluble polymers are preferred as the radiolysis of water is the dominant mechanism for the crosslinking of polymers to generate hydrogels [5]. The synthesis of hydrogels following irradiation with ionizing radiation leads to the formation of hydrogels with a homogeneous structure. The mechanical properties of E-beam crosslinked hydrogels are influenced by the irradiation dose and the proportions of polymers in the polymer blend [19,20].

Over the past few years, biocompatible hydrogels have garnered attention as scaffolds in TE [21,22,23]. Engineered tissue scaffolds are designed to mimic the unique composition and structural properties of the natural extracellular matrix, providing a supportive framework for cell survival and proliferation. Ideally, these scaffolds are biodegradable and capable of interacting with cells through specific bio-reactions that regulate cell adhesion and growth factors [22]. Furthermore, the new potential to solve a variety of TE issues, including tissue architecture, vascularization, and simultaneous seeding of many cells, has been made possible by the ability to regulate the surface, porosity, sizes, shape, and morphology of hydrogel scaffolds. Vascularization is necessary to facilitate the exchange of nutrients and the perfusion-based removal of waste products. There are two primary methods for promoting vascularization in a scaffold that has been tissue engineered. To encourage the vasculature from nearby host tissues to develop into the hydrogel scaffold, vasculogenic growth agents are added to the scaffold in the first method. In the second method, endothelial cells (ECs) are seeded onto the hydrogel scaffold [21]. Also, the design considerations for an effective hydrogel scaffold in TE received particular attention. However, some challenges using hydrogel scaffolds were reported [21].

This review article focuses on the role of ionizing radiation in the simultaneous synthesis and sterilization of polymeric hydrogels for potential applications in TE. A concise overview of ionizing radiation methods, including E-beam and γ-irradiation, is provided, highlighting their effects on crosslinking, polymerization, and grafting processes. This article also summarizes hydrogel systems fabricated using these technologies. Finally, conclusions, key outcomes, and future prospects in this field are discussed.

## 2. Types of Ionizing Radiation

Ionizing radiation technology is widely recognized for its dual role in hydrogel formation and simultaneous sterilization. This section provides a brief overview of two common forms of ionizing radiation: E-beam and γ-radiation.

### 2.1. E-Beam Radiation

E-beam irradiation involves the use of high-energy electrons produced by an accelerator. E-beam accelerators generate electrons with a wide range of energies, enabling the processing of various materials. Typically, electron accelerators with energies ranging from 0.3 to 100 MeV are used in diverse industrial applications. During the irradiation process, the accelerator produces a beam of energetic electrons. These electrons then interact with materials, changing their chemical and physical properties and ultimately enhancing the quality of the final product. Because an electron is a fundamental component of an atom, E-beam irradiation is considered a pure energy carrier. The interaction of accelerated electrons with polymers occurs in three distinct phases: physical, physicochemical, and chemical. During the physical phase, the primary electrons gradually transfer their energy to the polymer substrate, resulting in the formation of excited states, ions, and secondary electrons. This process generates short-lived reactive species [4].

Modern electron accelerators are highly versatile devices capable of producing beams with adjustable energy and power levels. The electron energy required for a specific application depends on the density and structure of the material to be irradiated and ultimately determines the most suitable type of electron accelerator. Accelerators are also classified based on beam power, which reflects their overall processing capacity. These devices provide variable energy for achieving different penetration depths and adjustable power for delivering low to high dose rates [24].

The Cockcroft‒Walton accelerator is a sophisticated piece of equipment widely used in scientific research and material development, including polymerization and biomaterial design. This device features a high-voltage generator with energy levels of 0.5 MeV and 2.0 MeV. Within the accelerator, electron beams are accelerated along both horizontal and vertical directions to accommodate various experimental requirements, achieving a maximum intensity of 30 mA for optimal performance. The accelerator beam can scan linearly across a width of up to 120 cm, ensuring uniform dose distribution. It is transferred from the vacuum environment into the air through a 50 µm-thick titanium window, which provides protection and maintains efficiency [25]. The MB10-30MP linear electron accelerator (Mevex Corp., Stittsville, ON, Canada) operates at an energy of 10 MeV and is utilized for high-energy electron irradiation. This device is equipped with a mobile platform featuring a maximum speed of 3 m/min and a scanning system with a frequency of 3 Hz, pulse length of 8 µs, and pulse repetition rate of 180 Hz [26].

The Rhodotron TT200 electron accelerator is tailored for applications requiring high doses or irradiation dose rates. It provides a nominal energy of 10 MeV, beam current of 4 mA, scan width of 100 cm, and scan frequency of 100 Hz. The accelerator also includes an adjustable-speed conveyor to transport calorimeters for dose measurement or other materials through the scanned beam. The distance between the accelerator’s exit window and the conveyor is approximately 120 cm [27].

The MT25 microtron in Prague is a cyclic electron accelerator equipped with a Kapitza resonator. Within this accelerator, electrons are accelerated by a radiofrequency electric field with a constant amplitude and frequency in the presence of a uniform and constant magnetic field. The electrons follow circular trajectories inside the vacuum chamber with a common tangent point. The energy of the accelerated electrons can be adjusted incrementally, ranging from 10 to 25 MeV in steps of approximately 1 MeV or from 6 to 12 MeV in steps of 0.5 MeV. Additional parameters include an electron current of 25 μA, a tunable magnetron frequency of 2790 ± 5 MHz, a peak power of 3 MW, a pulse length of 3 µs, a repetition rate of 425 s⁻^1^, a resonator frequency of 2796 MHz, and a power supply frequency of 50 Hz [28].

The electron linear accelerator ALID-7 is a Romanian pilot-scale device designed for fundamental research. ALID-7 has a vertical configuration to enhance the flexibility of beam processing, finding a wide range of applications in radiation processing. It supports the development of innovative E-beam-based technologies and offers commercial small-scale irradiation services, including monomer mixture polymerization, decontamination, and sterilization of medical products [29,30]. ALID-7, exhibiting an electron beam energy of 5.5 MeV, was originally designed to achieve peak E-beam currents ranging from 100 to 130 mA under variable pulse frequencies between 50 and 250 Hz, generating an average current of a few tens of microamperes. The current configuration sets the standard operating frequency at 53 Hz [31]. Furthermore, the irradiation duration, dose rate, and dose are continuously monitored and adjusted directly from the control desk in the accelerator control room.

E-beam irradiation induces physicochemical changes depending on the material, method, and applied dose. In turn, the physical properties of polymers can be precisely controlled by adjusting the irradiation dose and dose rate, providing optimal conditions for tissue healing. Compared to standard irradiation-induced crosslinking techniques, E-beam-assisted crosslinking can be considered a reagent-free approach [32].

### 2.2. Gamma Radiation

γ-rays are photons of electromagnetic radiation with energies ranging from 1 keV to 10 MeV and wavelengths between 10^−14^ m and 10^−10^ m. γ-rays are emitted by radioactive nuclei and can also result from interactions involving charged high-energy particles, such as high-energy electrons and photons capable of penetrating matter and initiating ionization. In addition to these pathways, γ-rays are also produced during nuclear reactions [33]. Their penetration characteristics resemble those of X-rays [34]. γ-radiation-induced crosslinking utilizes isotopes such as cobalt-60 (^60^Co) or cesium-137 to evenly crosslink polymeric chains and transform polymer solutions into hydrogels [35]. These isotopes emit high-energy photons (γ-rays) that can penetrate deeply into the target material [36]. The radiation dose, which refers to the amount of energy absorbed per kilogram of polymeric material, is measured in kiloGray [37].

High-energy γ-radiation induces crosslinking in polymers and modifies them, enhancing their thermal, mechanical, electrical insulation, environmental, and chemical properties. Owing to these favorable characteristics, their utilization in demanding applications across industries such as automotive, aerospace, defense, nuclear, and construction is increasing [38,39]. In biomedical applications, γ-radiation plays a critical role in synthesizing and modifying biopolymers for biocomposite fabrication [40]. Meanwhile, in hydrogel preparation, γ-radiation is a well-established technique for chemical crosslinking and TE device sterilization, valued for its safety and simplicity. This method eliminates the need for toxic crosslinkers or initiators, thereby removing additional extraction steps [41,42].

### 2.3. Comparison Between E-Beam and Gamma Irradiation Technology

The purchase of the E-beam accelerator or a γ-irradiator itself and the maintenance of the irradiation facility are the two primary costs associated with ionizing radiation processing. Auxiliary equipment, process monitoring, and control systems, the material handling system, building construction, and costs associated with acquiring suitable radiation shielding are all necessary in addition to the initial investment. In terms of these costs, it is necessary to budget for the obtaining of authorizations that comply with the requirements of the regulatory bodies that control nuclear-related activities.

The penetration power (depth penetration), throughput rates, the efficiency of radiation energy utilization, and the absorbed dose are all important technological factors for radiation crosslinking processes. Since the electron deposition is not constant, there is a position in the product that will receive the minimum delivered dose (Dmin), and another position that will receive the maximum dose (Dmax). The most important key parameter in industrial E-beam or γ-rays processing should be related to the dose uniformity ratio, known as the Dmax/Dmin ratio. For most technological irradiation processes, the accepted value is 1.35, but for sterilization purposes, this ratio usually has a lower value, around unity. Selecting the product thickness so that the dose on the opposite surface equals the dose on the front surface will result in the highest energy utilization efficiency. According to the characteristic energy deposition profile for a 10 MeV electron in water, for single-sided product irradiation with a minimum dose of 1.85 MeV cm^2^/g (1.85 kGy), having an optimum depth of 3.8 g/cm^2^, the effective absorbed energy is only 7 MeV; therefore, in this case, the utilization efficiency is about 70%. In these conditions, the optimal depth of E-beam material penetration depends on the beam energy and irradiation type. For practical reasons, E-beam industrial applications very often use double-sided product irradiation to increase the material thickness and better energy efficiency utilization. For single-sided irradiation with 5, 10, and 12 MeV, the optimal depth penetration will be approximately 1.7 cm, 3.3 cm, and 4.0 cm. For double-sided irradiation at the same beam energy, the penetration depth will be equal to 4.2 cm, 8.3 cm, and 10 cm, respectively. The E-beam throughput is directly proportional to the beam power and inversely proportional to the required dose. Higher beam power has the advantage of supplying higher throughput, which is very important for commercial production [43,44].

Radioactive ^60^Co sources are the most commonly used radiation source for TE radiation processing with γ-rays. The ^60^Co sources emit two photons with two energies of 1.173 and 1.333 MeV, respectively. The amount of radiation emitted by a radioisotope is expressed by the unit for radioactivity, Curie (Ci). At the industrial level, the power of 1 MCi of ^60^Co is equivalent to 14.8 kW, since 1 Ci of ^60^Co is equal to 0.0148 W. The low dose rate and absorbed dose by the matter per unit time of ^60^Co γ-irradiation is only 10^−3^ kGy/s. This is the main factor limiting the radiation processing throughput with γ-rays. The highest depth dose of ^60^Co γ-rays, which have an average energy of 1.25 MeV, is approximately 0.5 cm from the surface. When it comes to irradiating bulky objects with enormous volumes or irregular shapes, γ-irradiation has an advantage over E-beam irradiation due to its great penetration. The majority of industrial γ-processing facilities are involved for sterilization purposes of health care and disposable items, rather than polymer modification.

**Scheme 1:**
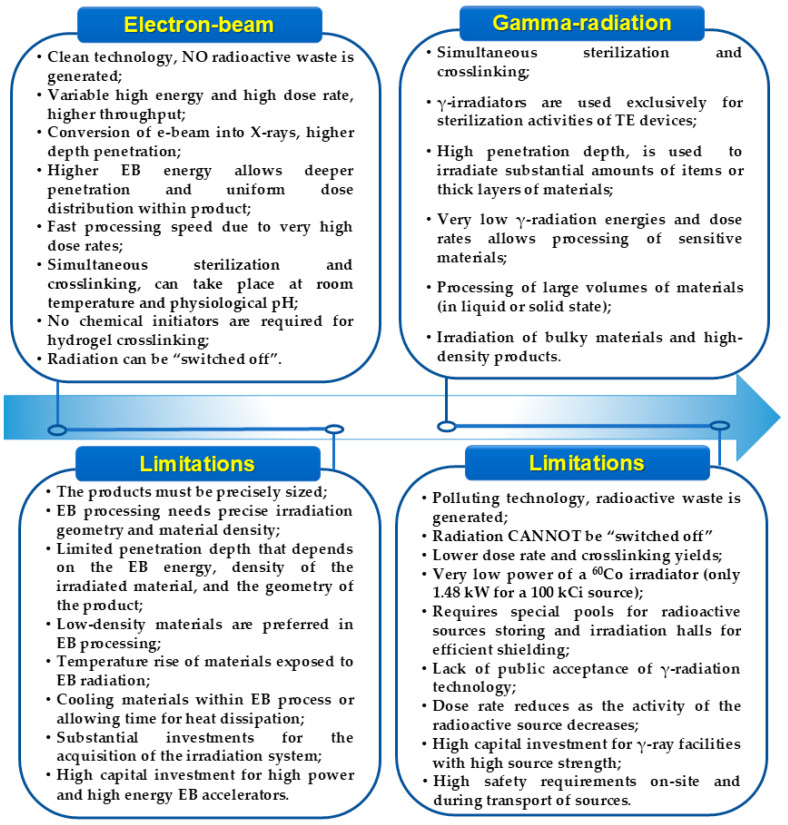
Advantages and limitations of E-beam and gamma irradiation technology.

It should be emphasized that building a new irradiation facility is not always the most economical way to use radiation technology in the industry due to high investment costs, particularly for small-scale production. Large-scale irradiation plants operate as service centers in different countries, offering irradiation on demand and commercial basis [45]. In Scheme 1, a schematic drawing to illustrate the use of different ionizing radiations is provided to compare the pros and cons of both techniques.

## 3. Radiation-Assisted Material Preparation Methods

Over the past few decades, extensive research has been conducted on the effects of ionizing radiation, such as E-beams and γ-radiation, on polymers [46]. This review aims to explore ways in which radiation can facilitate crosslinking, polymerization, and grafting processes in hydrogels for TE. These processes are typically conducted in aqueous environments and are based on the principles of radiation chemistry.

### 3.1. Radiation-Induced Crosslinking

Exposure to ionizing radiation in an aqueous polymer solution generates radicals on the polymer chains. The interaction of ionizing radiation with water generates various reactive species in diverse yields, with hydroxyl (·OH) and hydrogen (·H) radicals being the most predominant as indicated in Equation (1). The radiolytic yields of radicals depend on various factors, namely oxygen concentration in the aqueous polymer solution, absorbed radiation dose, polymer concentration, and polymer molecular weight. These factors in turn influence the efficacy of radiation-induced crosslinking. The fundamental mechanism of this crosslinking involves the abstraction of hydrogen atoms from the polymer backbone by ·OH and ·H radicals, leading to the formation of macroradicals (Equation (2)). The most stable macroradicals undergo free-radical recombination, forming stable covalent bonds between identical or different macroradicals. These covalent bonds provide structural stability to the final product (Equation (3)). In this context, RH can represent either a carboxymethyl chitosan (CM‒CHT) molecule or a gelatin (GEL) molecule [36]. The primary advantage of radiation-induced crosslinking lies in the elimination of chemical crosslinking agents, resulting in a cleaner and safer process. For successful crosslinking, the crosslinking reactions must predominate over chain degradation reactions [47,48]. However, under E-beam irradiation, the average molecular weight of polymers may decrease due to chain scission [49].

(1) $$H_{2}O\overset{\gamma radiation}{\to }\cdot OH,e_{aq}^{-},\cdot H,H_{2}O_{2},H_{2},H^{+},{OH}^{-}$$

(2) $$RH+\cdot OH\left(H\cdot \right)⇌R\cdot +H_{2}O(H_{2})$$

(3) R·+R·→R−R

### 3.2. Radiation-Induced Polymerization

**Scheme 2:**
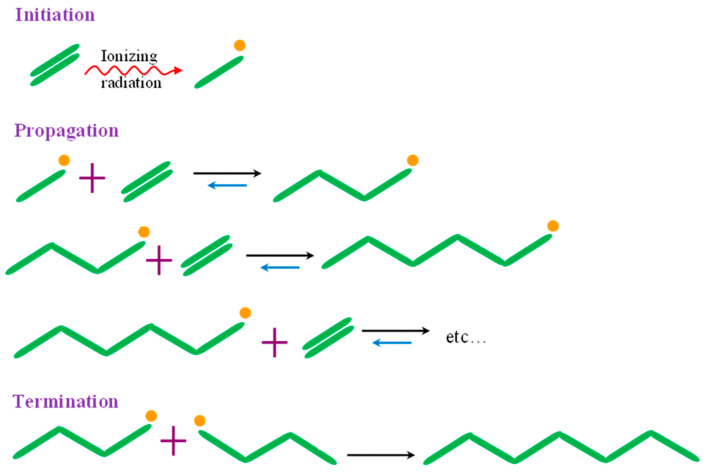
Radiation-induced polymerization [54].

Radiation-induced polymerization involves the generation of free radicals from monomers to create a network structure [50]. This polymerization technique is well established for various classes of monomers containing vinyl and vinylidene functional groups. Its advantages include the ability to synthesize polymeric structures with defined architectures, including macro- and nanostructures. Modern approaches to radiation-induced polymerization combine free-radical and ionic polymerization in a single step. These processes can be conducted under continuous radiation exposure, allowing control over the molecular weight of the final product [51]. The most commonly used monomers for synthesizing polymers in TE include acrylic acid (AA), acrylamide, methyl methacrylate (MMA), *N*-isopropylacrylamide (NIPAAM), hydroxyethyl methacrylate (HEMA), vinyl alcohol, vinylpyrrolidone, glycidyl methacrylate, and styrene [52]. Radiation-induced polymerization can be applied to monomers in various states—bulk, liquid, gas, or solid—as well as in suspensions or emulsions. In monomeric solutions, the polymerization process is initiated by solvent radiolysis, while the irradiation of bulk monomers generates ionized (radical cations) and electronically excited species. These species produce free radicals through neutralization, dissociation, or charge recombination. Ionization is the principal effect of energy loss from fast electrons, resulting in the formation of high-energy ions and electrons. Thermal electrons are subsequently generated and captured by radical cations (M·^+^) of monomers, forming excited molecules (M*) with energy levels exceeding the covalent bond energy of organic compounds. The resulted molecules participate in propagation reactions or dissociate into free radicals, depending on their energy states as shown in Scheme 2. Radiation-induced polymerization predominantly occurs via free-radical mechanisms and is influenced by the monomer’s characteristics, purity, and dose rate, along with reaction environment parameters [46]. During free-radical polymerization, the polymerization rate and the molecular weight of the polymer are proportional to the square root of the dose rate. Both quantities increase with temperature but are reduced by the presence of oxygen and other free-radical inhibitors [53].

### 3.3. Radiation-Induced Grafting

**Scheme 3:**
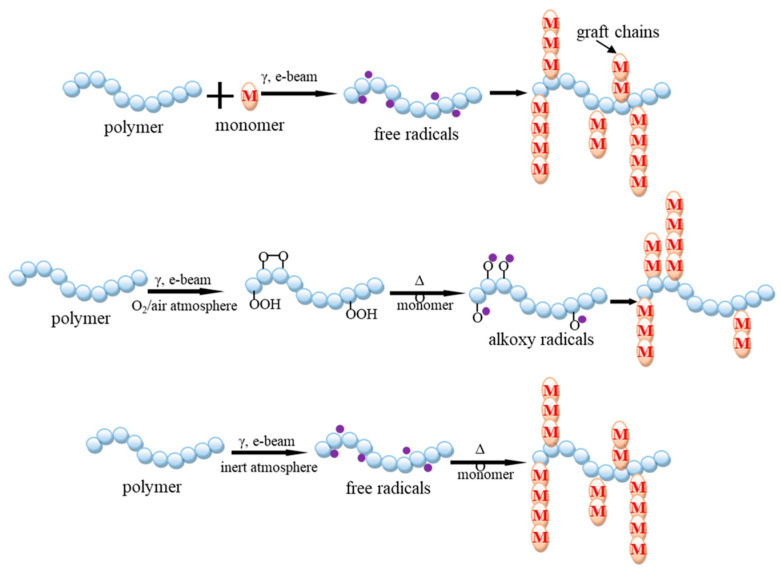
Radiation-induced graft polymerization [46].

Radiation-induced graft polymerization is an innovative technique for the surface modification of biomaterials. It enhances biocompatibility and customizes surface chemistry by immobilizing specific biomolecules onto blood-contacting biomaterials. During this process, the polymer is exposed to radiation in the presence of a monomer, which may be in vapor, liquid, or solution form. Irradiation can occur in air or, preferably, in an inert atmosphere to facilitate the generation of active free radicals in both the polymer and the monomer. These radicals interact rapidly, resulting in graft copolymerization as shown in Scheme 3. This method is considered highly efficient for grafting owing to the rapid reaction of the radicals; however, significant amounts of homopolymers may also form [55]. To address this, methods involving the addition of polymerization inhibitors to the monomer solution have been developed, significantly suppressing homopolymer formation. If a solvent or swelling agent is used, it must be carefully chosen to minimize the predominance of radicals generated from the solvent, which can again enhance homopolymer formation. Given the dependence of radiation absorption on material density, homopolymerization is generally minimal. Additionally, the radiation dose rate critically influences the process, as high dose rates can lead to rapid chain growth inhibition, reducing grafting yields and efficiency. This technique is particularly valuable for improving properties such as adhesion and biocompatibility [56]. γ-radiation-induced grafting is a preferred and efficient method for polymer modification and functionalization. It produces uniform active radicals, ensuring a high grafting yield without requiring catalytic contaminants or harmful initiators. In the biomedical field, γ-radiation is deemed ideal owing to its unique advantages [57].

### 3.4. Radiation-Induced Degradation

When matter and ionizing radiation combine, highly reactive species are created, including excited molecules, anionic and cationic ions, and free neutral radicals. These can significantly alter the irradiated material’s molecular structure. Organic polymers are specifically susceptible to molecular breakdown or scission, crosslinking, and molecular chain branching when exposed to radiation. Chain branching and crosslinking raise the polymer’s molecular weight. A network of insoluble three-dimensional polymers is created by crosslinking, whereas the original molecular weight is decreased via degradation or scission [58,59]. A small number of bonds in the polymer backbone can be broken, which can drastically affect the mechanical characteristics of the material [60]. All of these effects occur simultaneously during irradiation, and the frequency of each is influenced by several variables, including the polymer’s initial molecular structure and shape as well as the irradiation environment [61].

Ionizing radiation affects polymer materials differently, and certain materials are more likely to degrade or crosslink poorly when exposed to radiation. When exposed to radiation, certain natural or synthetic polymers may not crosslink well, while others may degrade significantly and lose their intended characteristics [53]. Understanding the fundamentals of radiation chemistry is necessary for the design and development of radiation-based hydrogel production systems that are effective, secure, and financially viable. This requirement has long been one of the primary drivers behind research into the radiolysis of polymers in aqueous solutions [62]. Material-specific challenges can be mitigated by designing novel polymers, blending materials, and utilizing radiation-resistant natural polymers.

## 4. Impact of Ionizing Radiation on the Formation of Polymeric Hydrogels for TE

Bone defects, such as those involving ligaments, tendons, cartilage, or irregular bone structures, are the primary targets of bone defect therapy. Autologous bone grafting is often regarded as a suitable option but is limited by the risks of lesion formation at the donor site and the scarcity of autologous bone. In this context, hydrogels in TE are promising candidates for bone repair scaffolds. They promote bone regeneration, adapt to irregular bone defects, and enable minimally invasive surgical procedures [63]. Ionizing radiation technology is a green, efficient, and environmentally protected fabrication method as compared to conventional preparation technologies [64]. Crosslinked hydrogels by irradiation allow cells to adhere and differentiate, thus simulating the natural tissue environment. They provide structural support in the affected areas, facilitating the repair of bone defects through internal healing mechanisms. As scaffolds used in bone TE, hydrogels must create an optimal environment for cells, ensuring water, oxygen and adequate diffusion of nutrients, as well as an appropriate pH and osmotic pressure, along with vitamins and minerals essential for cellular function. In addition, certain cells require sufficient space for proliferation, suitable attachment sites, and specific substrate properties, requirements that irradiated hydrogels can successfully fulfill [65].

Hydrogels are suitable for wound dressing and drug delivery applications owing to moisture retention characteristics and biocompatibility [66]. Hydrogels can be designed for controlled release, permitting drug release for a long time period and improved wound healing. Besides crosslinking, irradiation technology also provides sterilization of wound dressing, an essential prerequisite for dressing material which comes in contact with wounded skin. This technology ensures control over physical properties of the hydrogels with variation in polymer composition and absorbed dose [67].

### 4.1. E-Beam-Irradiation-Based Hydrogel Synthesis for TE

Numerous studies have explored E-beam-crosslinked hydrogels for TE applications. Table 1 summarizes various E-beam-irradiated polymer-based hydrogels and their corresponding outcomes in TE applications. Haema et al. reported that the molecular weight of GEL-based hydrogels decreased following radiation sterilization, yet their durability for biological and medical applications was maintained without the use of additional chemical agents. For instance, following exposure to doses of 5–25 kGy, the molecular weight of GEL decreased by 7–10%, while its stability was maintained for up to 7 days in water at 37 °C [68].

Haryanto et al. developed poly(ethylene glycol) dimethacrylate/poly(ethylene oxide) (PEO) and poly(ethylene glycol) dicarboxylate (PEGDC)/PEO hydrogels using E-beam radiation. The prepared hydrogels exhibited rapid swelling in water, stabilizing within 5 min. As the radiation dose increased from 100 kGy to 300 kGy, the swelling ratio decreased from 1468% to 744%, attributed to the enhanced crosslinking density. Following one week of incubation, cells exposed to PEGDC remained highly viable, demonstrating the extremely low toxicity of the PEGDC polymers to NIH3T3 fibroblast cells. The hydrogel composition containing 10% PEGDC exhibited a mechanical strength of 2130 kPa, suitable for biomedical applications. Additional properties included strong ductility, low toxicity, low hemolysis activity, and exceptional anti-adhesion barrier characteristics [69]. In another study, Haryanto et al. fabricated microporous hyperbranched poly(glycidol) (HPG)/PEO hydrogels using E-beam radiation. The pore size of the hydrogels could be adjusted by varying the HPG content in the polymer mixture. All hydrogel compositions demonstrated high cell viability after 7 days, and a significant increase in cell viability was observed for the 30% HPG hydrogel composition on the third day (*p* < 0.05). Cytotoxicity studies demonstrated that the HPG/PEO hydrogel scaffold effectively supported cell adhesion and proliferation. These findings suggest that crosslinked HPG/PEO hydrogels hold potential as scaffold materials in TE [70].

Riedel et al. explored the potential of high-energy E-beam-induced crosslinking to precisely tune collagen (COL) characteristics for extracellular matrix model systems. Quantification of the 3D pore size of the COL network as a function of radiation dose (10–100 kGy) revealed that increased crosslinking density reduced pore size. Cellular assays demonstrated that NIH3T3 fibroblast cells exhibited excellent cellular acceptance and well-maintained viability in the presence of the irradiated COL gels. Cultured fibroblasts on both unirradiated and irradiated COL exhibited approximately 95% viability, with 3% early apoptotic and 2% late apoptotic or necrotic cells, indicating no significant changes. Additionally, COL matrices demonstrated promise as highly tunable extracellular matrix mimetic systems with excellent cytocompatibility [71].

Cairns et al. subjected poly(L-lactic acid), poly(L-lactide-hydroxyapatite) (HAP), poly(L-lactide-glycolide) co-polymer, and poly(L-lactide-DL-lactide) co-polymer to low-energy E-beam irradiation. The study demonstrated that E-beam technology can enhance the performance of bioresorbable medical devices by enabling a depth-dependent degradation rate [84]. Krömmelbein et al. prepared agarose-based hydrogels using E-beam technology and evaluated their physicochemical properties. Rheological measurements revealed more than a 20% reduction in both storage (G′) and loss (G″) moduli at radiation doses of 30 kGy. Agarose crosslinked at doses up to 30 kGy exhibited sufficient robustness for biomedical applications, particularly in TE [74]. Mastalerz et al. investigated the effects of E-beam radiation on poly(lactic acid) and poly(caprolactone) (PCL) matrices combined with HAP, an inorganic filler found in natural bone. As the radiation dose increased, the mechanical properties of the polymer matrices deteriorated owing to chain scission. Despite this, the use of E-beam radiation for four-dimensional biomaterial printing has demonstrated considerable promise for bone TE, particularly in the development of biocomposite scaffolds and other medical applications [75].

Riedel et al. successfully incorporated alkaline phosphatase into GEL using E-beam irradiation to facilitate enzymatic mineralization with calcium glycerophosphate. Hydrogels containing alkaline phosphatase (1.25 and 2.5 mg/mL) demonstrated significantly higher uniaxial compressive moduli after E-beam irradiation at doses of 5–40 kGy compared to hydrogels without alkaline phosphatase. Therefore, mineralization improved the mechanical properties of the hydrogels. The cytocompatibility of the E-beam-crosslinked and mineralized GEL-based hydrogels was evaluated using proliferation assays, revealing good cytocompatibility. For instance, an evaluation of the interaction between the hydrogels and MG-63 human osteosarcoma cell lines over six days revealed good cell culture maintenance on all GEL hydrogel samples. These findings highlight the potential of E-beam-crosslinked and mineralized GEL-based hydrogels as thermally stabilized biomaterials for bone mimicry and replacement in bone TE [76].

Krömmelbein et al. produced granular agarose/alginate (ALG) hydrogels using electrospraying and E-beam irradiation. Their study involved a mechanical evaluation of the irradiated granular agarose/ALG hydrogels, which broaden the range of injectable hydrogels available for therapeutic applications. The mechanical properties of the hydrogels were preserved after injection, as evidenced by their self-healing ability. However, a decrease in the viscoelasticity of the granular hydrogel and individual granules was observed, attributed to radiolytic degradation under electron irradiation. Thus, the hydrogels subjected to higher radiation doses demonstrated limited applicability owing to the mechanical degradation of granules. The study also found that the relative polarity of the irradiation medium significantly influenced the chemical, mechanical, enzymatic, and biological properties of corneal tissue, based on ethanol concentration. Optimizing E-beam irradiation parameters at a dose of 25 kGy achieved tissue sterilization while maintaining long-term preservation at ambient temperature. After seven days of culture, human corneal stromal cells grown on irradiated corneas exhibited cell viability greater than 93%, comparable to those cultured on tissue culture plates (approximately 92%). Similarly, human corneal epithelial cells grown on irradiated tissues demonstrated viability above 91%, comparable to the viability of 95% for those grown on tissue culture plates after seven days. Irradiation did not affect the biocompatibility of the corneal transplant, as all samples exhibited confluence, a spreading structure, and a morphology similar to tissue cultures [77].

In a separate study, Ling et al. used pre-irradiated polypropylene sheets treated with E-beam irradiation to successfully prepare poly(acrylamide-co-acrylonitrile) through graft polymerization. The hydrogel surface exhibited optimal swelling behavior at 32 °C. CCK-8 assays revealed no apparent cytotoxicity of the hydrogels against the 293T cell line. These smart hydrogels demonstrate potential applications in TE and cell harvesting [79].

**Figure 1:**
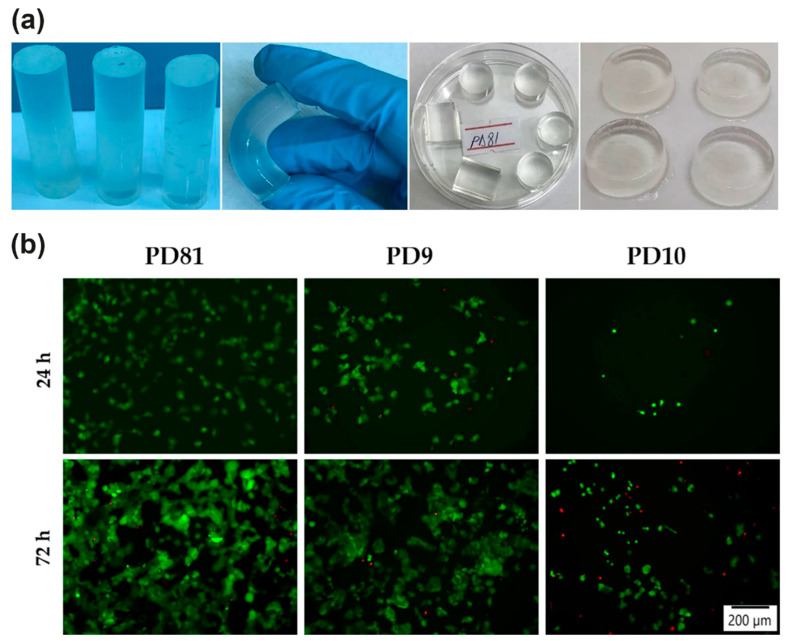
(**a**) Multi-component polymeric hydrogels fabricated via electron (E)-beam irradiation. (**b**) Fluorescence images of hydrogels after 24 h and 72 h of cell seeding, obtained from the LIVE/DEAD assay (red fluorescence indicates dead cells; green fluorescence indicates living cells). Reproduced with permission [80]. Copyright 2023, MDPI.

In various studies, Demeter et al. prepared multi-component hydrogels composed of bovine COL, poly(vinyl pyrrolidone) (PVP), PEO, carboxymethylcellulose (CMC), and CHT using E-beam irradiation, as illustrated in Figure 1a. The hydrogels exhibited rheological behavior typical of soft solids, with G′ values ranging from 9.69 to 14.98 kPa, and demonstrated good stability in a swelling environment. In vitro tests on Vero cells, conducted through both indirect and direct contact, revealed exceptional biocompatibility of the samples, as depicted in Figure 1b. However, quantitative CCK-8 analysis indicated that hydrogels containing CHT resulted in lower cell viability compared to the other hydrogels, suggesting an inhibitory effect on cellular metabolic activity or restricted proliferative capability [80]. Similarly, Demeter et al. developed biocompatible hybrid hydrogels with good elastic characteristics via E-beam-induced crosslinking, using CHT, water-soluble polymers (PVP, PEO), AA, and commercial silver nanoparticles (AgNPs). These composite hydrogels exhibited elasticity, flexibility, biocompatibility, and antimicrobial activity against common pathogens found in infected wounds. The properties of the hydrogels were significantly influenced by the presence of AgNPs and the absorbed radiation dose [83]. In another study, Demeter et al. utilized E-beam-induced crosslinking to produce highly elastic and superabsorbent hydrogels composed of COL/PVP/poly(acrylic acid) (PAA)/PEO quaternary copolymers for soft TE. The hydrogels displayed solid-like behavior comparable to that of soft tissues, including human skin, with G′ values ranging from 4500 to 14,000 Pa. The hydrogel’s maintained stability and physical integrity for approximately 50 h under conditions simulating both healthy skin (pH 7.4) and infected wounds (pH 9.4) [72].

**Figure 2:**
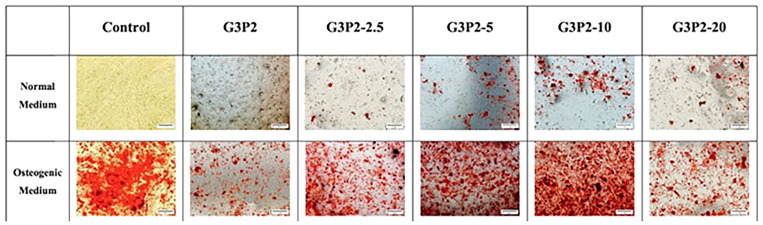
Qualitative evaluation of the mineralization and calcium deposition of rabbit-bone-marrow-derived mesenchymal stem cells on tragacanth gum/poly(vinyl alcohol)/halloysite nanotube hydrogels after up to 21 days of incubation. Reproduced with permission [81]. Copyright 2023, Elsevier.

Dehghan-Niri et al. synthesized tyramine (TA)-loaded tragacanth gum (GT) and poly(vinyl alcohol) (PVA)-based hydrogels using E-beam irradiation. Subsequently, they evaluated the effects of PVA on the hydrogels’ properties. The results indicated that increasing the PVA ratio and irradiation dose to 28 kGy significantly enhanced gel content, mechanical strength, viscoelastic behavior, and elastic recovery. After 168 h of incubation, the hydrogels demonstrated relative cell viability values above 92%, with no significant differences (*p* > 0.05). Results from MTT and LIVE/DEAD assays confirmed that the hydrogels were biocompatible and non-cytotoxic. These findings indicate the potential application of these hydrogels in biomedical settings, particularly for cartilage TE [73]. In a subsequent related study, Dehghan-Niri et al. developed nanocomposite hydrogels composed of TA-conjugated GT/PVA/halloysite nanotubes (HNTs) using E-beam irradiation at 28 kGy. The nanocomposite hydrogels displayed enhanced mechanical properties, with a fracture stress of 371.82 kPa and a gel content of 88.2% at a 10% HNT concentration. Biocompatibility and osteogenic activity were evaluated by seeding rabbit-bone-marrow-derived mesenchymal stem cells (rBMSCs), as illustrated in Figure 2. After seven days of cultivation, cell viability exceeded 85%, indicating low cytotoxicity of the hydrogels. A notable increase in cell viability was observed in the nanocomposite hydrogels containing 5–10% HNTs, attributed to improved cell adhesion and proliferation, which resulted in enhanced biological activity of rBMSCs. These findings suggest that nanocomposite hydrogels have significant potential for bone TE applications [81].

Sramkova et al. utilized E-beam irradiation to enhance the biocompatibility and in vivo efficacy of implantable devices through non-biofouling hydrogel coatings based on poly[(2-methyl-2-oxazoline)-co-(2-(3-butenyl)-2-oxazoline)]. The hydrogels exhibited uniform smoothness and consistent shape, regardless of polymer type or irradiation dose. Elasticity coefficients ranged from 500 to 1200 kPa and increased with the radiation dose. Cell adhesion was more significantly affected in hydrogels produced from a 1 wt% copolymer solution, likely due to insufficient crosslinking and structural irregularities in the hydrogel network. In contrast, hydrogels prepared from a 5 wt% copolymer solution demonstrated excellent non-biofouling properties. These findings indicate that hydrogel stiffness, determined by the degree of crosslinking, can be optimized for specific applications in living tissue [82].

### 4.2. Gamma-Irradiation-Based Hydrogel Synthesis for TE

Ionizing radiation, such as γ-radiation, has been demonstrated to be effective for the sterilization and crosslinking of polymeric films while ensuring the absence of toxic byproducts [85]. Sterilizing hydrogels is crucial for meeting practical clinical requirements. Table 2 summarizes various studies utilizing γ-irradiation and its role in TE. Gujjar et al. fabricated natural hydrogels using enzymes and detergents through the perfusion-based decellularization of human placental tissue, followed by the generation of placental tissue powder-based extracellular matrix (P-ECM) [86]. Hydrogels were subsequently prepared with and without γ-sterilization. Biological evaluations, including cell viability and proliferation assays, along with physicochemical characterizations, demonstrated that the P-ECM hydrogels were biocompatible and exhibited improved mechanical strength. These findings suggest that P-ECM-based hydrogels demonstrate potential as adaptable platforms for 3D culture systems, accelerating the development of effective TE techniques for soft tissue regeneration and rejuvenation. Similarly, Na et al. prepared an ECM-hydrogel-incorporated microfluidic chip to serve as a 3D blood vessel model, evaluating the effects of γ-sterilization on its angiogenic and endothelial barrier functions [87]. Human dermal microvascular endothelial cells (hMVECs) were seeded into the 3D blood vessel model and exposed to γ-sterilization after four days, followed by additional culturing for another four days post-irradiation. Observations revealed reorganization in the fiber structure, an increase in apoptotic cells, and a reduction in tight junction protein Claudin 5 in γ-exposed hMVECs. In another study, bone-mimicking structures were created by incorporating magnesium-substituted calcium phosphate ceramics into PVA hydrogels, while cartilage-mimicking structures were developed by integrating high-performance nanofiber reinforcements (Zylon^®^ and Kevlar^®^) into PVA [88]. Both structures underwent γ-sterilization (25 kGy at 5 kGy h^−1^) to eliminate undesirable effects. The resulting bone- and cartilage-mimicking structures closely resembled natural tissues, meeting essential conditions such as those for liquid content, porosity, and biological behavior. Thus, these structures hold promise for addressing osteochondral defects. Additionally, Dey et al. investigated the effects of γ-sterilization on the thermal and physicomechanical properties of various hydrogels, including GEL/polyethylene glycol (PEG), GEL/PEG/hydroxyethyl cellulose (HEC), and GEL/PEG/CHT [89]. The results revealed that γ-sterilization had negligible impact on the stress relaxation, mechanical properties, and water uptake of GEL/PEG and GEL/PEG/CHT hydrogels. However, GEL/PEG/HEC hydrogels were found to be more vulnerable to irradiation-induced effects.

In a study conducted by Anderson et al., ALG foams were prepared from aerated ALG solutions using the ionotropic gelation method, followed by air drying [113]. These air-dried foams were then exposed to γ-irradiation, which reduced the molecular weight of ALG and decreased the foam strength without altering the foam’s appearance. Characteristics of the dried ALG foams including pore interconnectivity, average pore size, mechanical properties, and absorbency were influenced by factors including the type and concentration of ALG, the amount of incorporated air, and the γ-irradiation dose. To optimize ALG foam preparation for specific applications, such as wound dressing, parameters like dry foam density, ALG concentration, and alginate type can be adjusted to achieve adequate fluid drainage, maximum absorbency, and mechanical integrity, enabling one-piece removal.

In comparative studies, researchers evaluated the effects of various sterilization techniques on the properties of hydrogels. Kanjickal et al. examined the impact of hydrogen peroxide (H_2_O_2_), ethylene oxide, and γ-sterilization on PEG hydrogels for TE and controlled drug delivery [34]. γ-sterilization increased free-radical concentrations, reduced swelling fractions, and decreased surface roughness. While γ-sterilization altered the release profile of the immunosuppressive agent cyclosporine A (CyA), the release of rhodamine B remained unchanged. Castellanos et al. synthesized PEG, multi-arm PEG, and CHT-PEG hydrogels using free-radical polymerization, followed by either 70% disinfection or γ-sterilization [106]. Fourier-transform infrared spectroscopy analysis revealed shifts in spectroscopic bands to lower or higher energies with varying intensities. Ex vivo hemocompatibility assays, including platelet aggregation, hemolysis, and mononuclear cell viability and morphology, along with biocompatibility studies in human fibroblasts, confirmed the hydrogels’ cytocompatibility and hemocompatibility. These findings suggest the potential of these hydrogels for skin TE applications. Similarly, Peng et al. developed poly-(2-hydroxyethyl methacrylate) (pHEMA), AA/pHEMA, AA/pHEMA/iron (Fe^3+^)-based hydrogels and tested them using various sterilization techniques, including γ-sterilization at 25 kGy [94]. Unsterilized samples exhibited mold growth when stored at 4 °C. Irradiation sterilization caused slight changes in the intensity of the hydroxyl and carboxylic groups, as well as random bond cleavage due to scission reactions. These results demonstrate that optimized sterilization methods can address clinical requirements and expand the applicability of hydrogels in biomedical fields, including TE.

Rizwan et al. investigated the effects of various FDA-approved sterilization methods, including γ-sterilization, on GEL methacryloyl (GelMA) bioink and hydrogels prepared from GEL [96]. γ-sterilization resulted in reduced pore size, increased stiffness, a slower biodegradation rate, inhibited sol‒gel transition, and decreased printability. However, none of the sterilization methods affected the 2D spreading and adhesion of endothelial or fibroblast cells. Hence, GelMA matrices with slower degradation and increased stiffness, induced by γ-irradiation, were deemed suitable for TE applications. In another study, Merchan et al. prepared scaffolds for TE containing ALG, NC, and hyaluronic acid (HA) using 3D printing [95]. The bioinks were sterilized using autoclave, β-radiation, and γ-radiation procedures to make them compatible with mesenchymal stromal cells (D1-MSCs). Biological analysis revealed that sterilization enhanced biocompatibility and resulted in higher cell viability against D1-MSCs. Overall, this study demonstrated that the prepared bioinks are promising candidates for regenerative medicine and TE applications.

Irradiation techniques are well-established, clean, and sterile methods for hydrogel development, suitable for various biomedical applications. These methods allow for simultaneous sterilization and gel formation [117,118]. Several studies have explored the potential of γ-irradiation-crosslinked hydrogels for tissue regeneration and bone, cartilage, and vascular TE. For instance, El-Hag Ali et al. demonstrated the in-situ formation of nano-HAP (nHAP) through γ-irradiation-induced crosslinking and copolymerization to prepare nHAP/GEL/AA-based nanocomposites [112]. Physicochemical analysis confirmed the formation of nHAP, while clot formation ability and hemolysis studies verified the biocompatibility of the developed composites. These findings suggested the potential of the nanocomposites as materials for bone TE. Similarly, Jeong et al. prepared electrospun PCL-based nanofibers, immersed them in a graphene oxide (GO)-containing PVP solution, and exposed them to γ-irradiation (25 kGy/h) to fabricate GO/PVP/PCL nanofibrous scaffolds [93]. The scaffolds were loaded with bone morphogenetic protein-2 (BMP-2), a bone regeneration growth factor, and subjected to electrical-stimulation-based release testing. The hydrogels demonstrated osteodifferentiation, excellent mineralization, and BMP-2 release, highlighting their potential for bone TE applications. Chen et al. developed biodegradable and temperature-responsive scaffolds composed of glycidyl methacrylated dextran (DEX) and GEL containing BMP-2-loaded microspheres through radical crosslinking and low-dose γ-irradiation [116]. The microsphere scaffolds exhibited biodegradability in phosphate-buffered saline, with the presence of DEX enabling adjustable degradation rates. BMP release at 22 °C and 37.2 °C occurred in a controlled manner, with the release period ranging from 18 to 28 days. This study demonstrated the feasibility of integrating cell/tissue scaffolds with drug delivery carriers into a single device. Furthermore, it revealed that the new, easy-to-use scaffolds could help effectively retain the biological activity of growth factors while sustaining therapeutic concentrations during the healing process.

Raafat et al. synthesized magnesium-doped nHAP/Acacia gum-GEL-based scaffolds for bone TE using γ-irradiation [98]. The prepared hydrogels exhibited excellent antibacterial activity against *Escherichia coli* (*E. coli*) and *Staphylococcus aureus* (*S. aureus*) and demonstrated sustainable Ketoprofen release. In vitro blood compatibility and cytotoxicity analyses confirmed their biocompatibility, validating these hydrogels as candidates for bone TE applications. Similarly, Kim et al. fabricated silk fibroin (SF)/HAP-nanoparticle-based composite hydrogels using γ-irradiation for bone TE [104]. Morphological analysis revealed a highly porous structure in the developed hydrogels. The SF/HAP composite hydrogels enhanced osteogenic differentiation and improved cell adhesion and proliferation, highlighting their potential as biomaterials for bone TE applications. Daly et al. developed MSC-laden, aspartic-acid (RGD)-functionalized-γ irradiated ALG 3D-bioprinted hypertrophic cartilage models for whole bone organ engineering [105]. These models were reinforced with PCL fibers, enhancing their compressive modulus by up to 350 times. Using this approach, vertebral bodies with trabecular-like bone, functional vasculature, and supporting marrow cavities were successfully developed. The findings suggest that these models hold significant potential for whole bone development in craniofacial medicine and orthopedics.

For cartilage TE, Critchley et al. engineered cartilage models by incorporating BMSCs into arginine-glycine-RGD-functionalized, γ-irradiated Alg hydrogels for osteochondral (OC) defect regeneration [101]. The models were analyzed using computational modeling, micro-computed tomography, biochemical assays, mechanical testing, cartilage template implantation, histology, immunochemistry, and histological scoring. The findings indicated enhanced OC defect regeneration in the developed cartilage models, demonstrating the capacity of these BMSC-incorporated soft tissue models to regenerate diseased and damaged joints. Similarly, Zhou et al. fabricated decellularized cartilage matrix (dECM)/SF-based hydrogels using γ-irradiation at a total dose of 60 kGy at 196 Gy/min at room temperature [17]. The hydrogels exhibited excellent water absorption capacity and biocompatibility. In vivo studies confirmed that their multi-channel structure and dECM components were conducive to the chondrogenic differentiation of adipose-derived stromal cells. These findings indicate that the prepared hydrogels are promising candidates for cartilage TE applications.

For vascular TE, Shin et al. developed biomimetic dual-layered scaffolds in which poly(L-lactide-co-ε-caprolactone) was electrospun into nano- and microfibers to form the basic vessel structure [111]. The layered structure was functionalized using γ-irradiation, along with GEL immobilization through AA. The functionalized microfibrous layer enhanced smooth muscle cell (SMC) proliferation and infiltration, while the nanofibrous layer regulated human umbilical vein endothelial cell adhesion and proliferation through cell-to-cell interactions. This layered and radiation-modified scaffold effectively mimics native blood vessels, making it a promising candidate for vascular TE and biomedical applications.

For skin TE, Zhao et al. fabricated scaffolds using HA/chondroitin sulfate (CS)/PVA [109] and HA/CS/PAA [107] via γ-irradiation. The hydrogels exhibited high gel fractions and water content (over 90%). Their enzymatic degradation kinetics depended on the hydrogel composition and the hyaluronidase solution. Theophylline and cefazoline were used as model nonionic and ionic drugs for in vitro drug release analysis, with significant drug release observed in both cases. When cultured with human keratinocyte cells, the scaffolds demonstrated high cell viability (above 80%). These findings indicate that the prepared scaffolds have strong potential for skin TE applications.

For tissue regeneration, González-Torres et al. investigated the biological activity of γ-irradiation-crosslinked COL-PVP-PEG hydrogels [102]. Physicochemical analysis confirmed the bonding and morphology of the developed hydrogels, while cytotoxicity assays showed higher fibroblast cell line viability at lower concentrations of PVP/PEG. These findings present a viable methodology for developing 3D terpolymer hydrogels for cell attachment, making them suitable for connective tissue regeneration applications. Similarly, Zhang et al. developed COL/DEX composite hydrogels using γ-irradiation [110]. The composite hydrogels exhibited enhanced hydrophilicity, a porous structure, higher water uptake, reduced crosslinking density, and lower stiffness compared to COL-only scaffolds. In vivo and in vitro analyses confirmed excellent degradation, along with significant histocompatibility and cytocompatibility. These properties make the composite hydrogels ideal for inducing tissue regeneration and repairing dermal defects.

**Figure 3:**
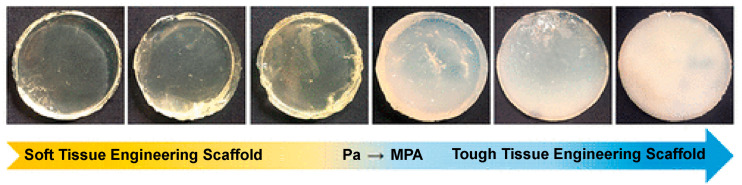
Appearance of silk-fibroin-based soft and tough tissue engineering scaffolds. Reproduced with permission from [97]. Copyright 2020 American Chemical Society.

Several studies have demonstrated the potential of hydrogels for TE and various biomedical applications. For instance, Wu et al. fabricated SF hydrogels through γ-irradiation-induced crosslinking at various doses and studied its impact on the mechanical and structural properties of the hydrogels [97]. Images of the SF-based soft and tough TE scaffolds are presented in Figure 3. γ-irradiation created stable and uniform active sites on the SF for intra- and inter-molecular interactions. The prepared hydrogels exhibited tunable porosity, a uniform pore structure, controlled degradation time, adjustable mechanical strength, and good biocompatibility with rat BMSCs, making them suitable for TE applications. Similarly, Mahdy et al. copolymerized zein and poly(vinyl butyral) (PVB) to prepare a polymeric blend-based scaffold using γ-irradiation [91]. The process improved adhesion between zein and PVB and enhanced the thermal stability of the scaffold. Vero (monkey kidney) cells proliferated more effectively on the scaffold irradiated at 45 kGy compared to unirradiated samples, indicating its suitability for TE applications. Zhang et al. prepared COL-based hydrogels through γ-irradiation [108]. The hydrogels were then submerged in a complete medium (Dulbecco’s Modified Eagle Medium with 10% fetal bovine serum and 1% antibiotics) and evaluated for compressive mechanical properties. Contractile COL hydrogels were cultured with fibroblast cell lines for biological analyses, including cell viability, cell proliferation, apoptosis, morphology, stress fiber formation, and cell cycle studies. The results revealed accelerated apoptosis and increased L929 cell proliferation, making these hydrogels promising for TE applications. Lee et al. developed poly(γ-glumatic acid) scaffolds using γ-irradiation and their apatite composites through a soaking method for cell proliferation studies [114]. Various physicochemical analyses confirmed the successful development of the scaffolds and their composites. Biological analysis revealed excellent MC3T3-E1 cell proliferation after seven days of culture on the composite scaffolds, indicating their safety and efficacy as biopolymers for in vivo and in vitro TE applications.

**Figure 4:**
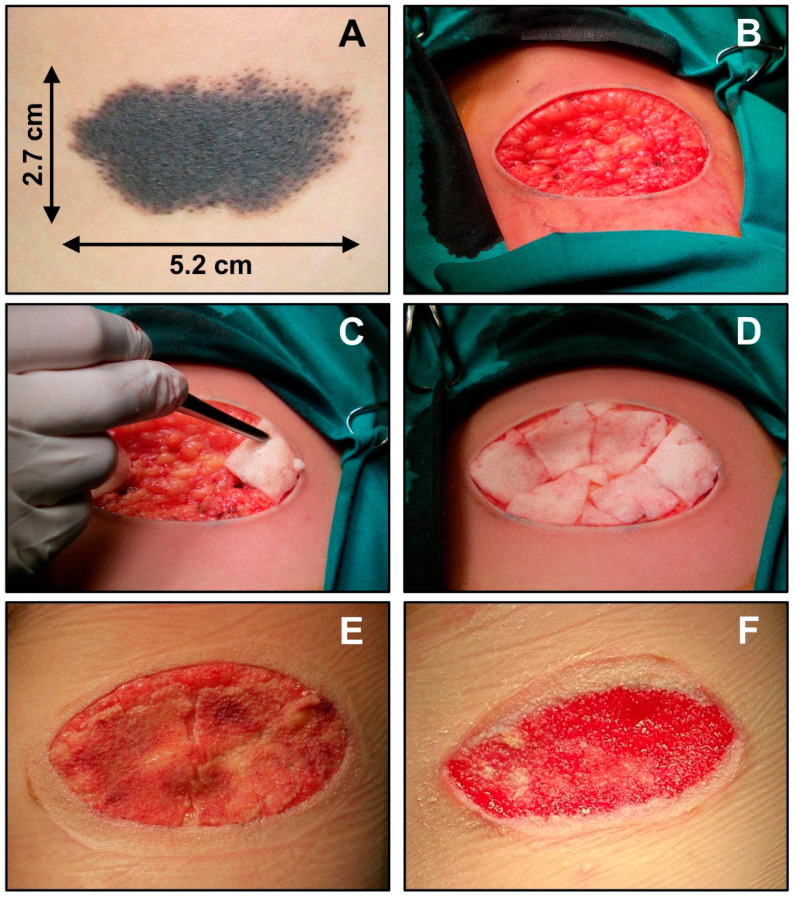
Human clinical trial outcomes of γ-irradiation-sterilized wound dressing applied to a nevus resection wound measuring 5.2 × 2.7 cm^2^ over 4 weeks. (**A**) Congenital melanocytic nevus, (**B**) skin wound post-surgical resection, (**C**) grafting procedure using small dressing pieces, (**D**) complete wound coverage by the dressing, (**E**) early biodegradability observed after seven days, and (**F**) early regeneration observed after four weeks. Reproduced with permission from [103]. Copyright 2017 Elsevier.

Weinstein-Oppenheimer et al. developed GEL/CHT/HA-based porous scaffolds using freeze-drying, followed by γ-irradiation for sterilization [103]. The scaffolds were modified with calcium chloride and autologous plasma to create clot scaffolds. Human mesenchymal stem cells were subsequently incorporated into the porous scaffolds to create biomaterial-based wound dressings. In vivo assays revealed excellent wound healing capabilities of the dressings. Figure 4 illustrates the outcomes of clinical trials where a γ-irradiated wound dressing was applied to a nevus resection wound measuring 5.2 × 2.7 cm^2^ for 4 weeks. The clinical studies confirmed the biosafety of the developed dressing. Biological analyses demonstrated partial biodegradation at one-week, high biocompatibility, early regeneration capacity at four weeks, and no signs of rejection.

In another study, Yue et al. fabricated 3D cellulosic scaffolds using a temperature-responsive phase separation technique facilitated by γ-irradiation, creating a crosslinked structure suitable for soft TE [115]. Biological analyses and biocompatibility tests showed minimal inflammatory responses up to 12 weeks of subcutaneous implantation in mice and cytocompatibility across various cell types. The scaffolds exhibited high water content, interconnected macroporosity, mechanical integrity, and cytocompatibility, making them suitable for soft TE applications. For wound dressing applications, Singh et al. incorporated silver nitrate into polymeric blends of CMC/PVP, exposing them to γ-irradiation to create antimicrobial skin dressings for burn wound management [92]. The hydrogels demonstrated fluid absorption, moisture transmission, and impermeability to microbes. Antimicrobial efficacy was observed against *S. aureus*, *Pseudomonas aeruginosa*, *E. coli*, and *Candida albicans*, attributed to the integration of AgNPs. These dressings are effective for treating and preventing microbial infections in burn injuries. Singh et al. prepared gentamicin-loaded AG/TG-based hydrogels for wound dressing using γ-irradiation [99]. The prepared hydrogels exhibited mucoadhesiveness, antioxidant and antibacterial activity, impermeability to microorganisms, and permeability to oxygen and water. These properties demonstrate the potential of the hydrogels for wound healing applications. Barriguete et al. synthesized temperature-responsive hydrogels based on the copolymers of NIPAAm, dimethyl acrylamide (DMAAm), and MMA, as well as the copolymers of NIPAAm, DMAAm, and ethoxyethyl methacrylate, using γ-irradiation [85]. These smart hydrogels displayed biocompatibility, thermo-responsiveness, conformability, and durability, making them ideal candidates for intelligent polymer applications, including smart membranes, biological sensors, and flexible electronics.

**Figure 5:**
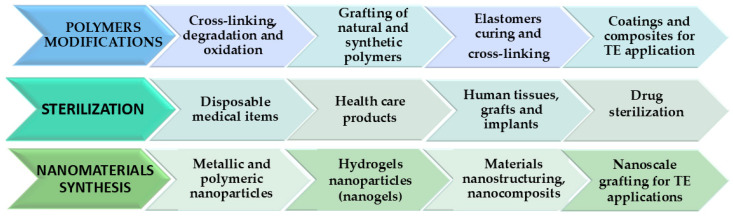
The potential applications of the use of ionizing radiation in TE applications.

Ionizing radiation is used for many beneficial applications, including uses in medicine, research, industry, the environment, and security. Figure 5 shows the applications for ionizing radiation in TE applications.

### 4.3. Regulatory Considerations and Clinical Translation of Radiation-Synthesized Hydrogels

Degradable scaffolds can benefit traditional biomedical applications, such as joint replacement, which currently use non-degradable metallic scaffolds. A scaffold that promotes bone or cartilage regrowth and has adequate mechanical properties can reduce the need for revision surgery in joint replacements [119]. Before using a material, its biodegradability must be considered. In TE, the ideal is for the scaffold to decompose and the host tissue to regenerate. The degradation rate of the material can be adjusted to match the rate of regrowth [120].

Hydrogels have been employed in clinical trials for a while, which is consistent with their many biological applications. This is not to suggest, however, that hydrogels’ clinical translation is straightforward or trivial. Even though a substantial infrastructure has been established to consistently create more conventional hydrogels (such as covalent gels) for use in bandages to contact lenses, the emergence of hydrogels can be far more complicated from a physical and chemical perspective. When attempting to adhere to the federally mandated Quality System Regulations (QSRs) and Current Good Manufacturing Practices (cGMPs), these complexities may provide difficulties [121]. The variety of biomaterials and crosslinking mechanisms used to fabricate hydrogels makes it difficult to classify and approve them for regulatory purposes. The hydrogels fall within the “devices” category, according to Section 201(g) of the FD&C Act, which covers “any product which does not achieve its primary intended purposes through chemical action within or on the body”. A 510(k) Pre-Market Notification filing must be reviewed by the FDA for the majority of hydrogel-based products to receive legal marketing rights in the US, with very few exceptions. Hydrogels containing drug or drug-secreting cells are regarded as combination products, and as such, they need up to seven to ten years for regulatory clearance, which further restricts their economic viability [122].

## 5. Conclusions, Challenges, and Future Prospects

Ionizing radiation offers a promising method for hydrogel fabrication in biomedical applications, enabling initiator- and crosslinker-free synthesis of highly pure hydrogels under mild conditions, along with simultaneous sterilization. However, optimizing radiation parameters is crucial for advancing the technology and developing robust hydrogel systems. The swelling degree and gel fraction of hydrogels can be adjusted by regulating the radiation dosage during fabrication. Higher doses may lead to reduced pore size, excessive crosslinking, and polymer chain degradation, whereas lower doses may result in inadequate structural strength and insufficient crosslinking. Therefore, determining the appropriate radiation source, dose, dose rate, distance, energy, current, and environmental conditions is essential. The integration of bioactive agents or nanomaterials into hydrogels can enhance their performance, provided that the radiation dose is carefully optimized to achieve effective crosslinking without compromising structural integrity. Preclinical and clinical trials are necessary to evaluate the efficacy and sterility of hydrogels fabricated under ionizing radiation exposure for biomedical applications. Advancements in this environmentally friendly fabrication technology can significantly expand the use of ionizing-radiation-developed scaffolds, making them viable for a wide range of biomedical applications.

## Figures and Tables

**Table 1:** Raw materials, fabrication technologies, applications, and outcomes of various studies. TE refers to tissue engineering.

Polymers	Ionizing Technology	Applications	Findings	References
GEL	E-beam; acceleration energy: 2 MeV; beam current: 2 mA; doses: 10, 20, 40, 60, 80, and 100 kGy; dose rate: 10 kGy/pass	Biological and medical applications	High gel fraction, efficient crosslinking, good swelling ability and stability	[68]
PEGDMA, PEO, PEGDC	E-beam; acceleration energy: 0.7 MeV; beam current: 5 and 10 mA; doses: 100, 150, 200, 250, and 300 kGy	Biomedical materials (anti-adhesive barriers)	Good biodegradability, excellent mechanical properties, adequate tissue adherence, low hemolysis capability, low toxicity	[69]
HPG, PEO, potassium methoxide, glycidol	E-beam; acceleration energy: 0.7 MeV; beam current: 5 and 10 mA; dose: 300 kGy	TE	Suitable water absorption capacity, good cell viability, low cytotoxicity	[70]
COL	E-beam; acceleration energy: 10 MeV; pulse repetition: 180 Hz; doses: 10, 20, 40, 60, and 100 kGy; dose rate: 5 kGy/min	TE, mammalian extracellular matrix-mimetic systems	Adequate mechanical properties, crosslinked structure, excellent cytocompatibility, cell viability	[71]
COL, PVP, PAA, PEO	E-beam; acceleration energy: 6 MeV; beam current: 10 μA; pulse repetition: 53 Hz; doses: 15, 20, and 25 kGy; dose rate: 4 kGy/min	TE, wound dressing	Excellent mechanical properties, permanent network structure, swelling capacity, stability	[72]
TA, GT, PVA	E-beam; acceleration energy: 10 MeV; doses: 14, 28, and 56 kGy; room temperature	TE	Higher crosslink density, interconnected pores, excellent elastic recovery, non-cytotoxicity	[73]
Agarose	E-beam; acceleration energy: 10 MeV; pulse repetition: 180 Hz; doses: 5, 10, 15, 20, 25, and 30 kGy	TE	Viscoelastic properties, degradation at high electron doses	[74]
PLA, PCL	E-beam; acceleration energy: 10 MeV; doses: 10, 15, 25, 50, 75, and 100 kGy; dose rate: 15 kGy/s	Bone TE	Adequate structural characteristics, stable network structure	[75]
GEL, alkaline phosphate, calcium glycerophosphate	E-beam; scanning frequency: 3 Hz; pulse repetition: 180 Hz; pulse length: 8 μs; doses: 5, 10, 15, 20, and 40 kGy	Bone TE	Good stability, increased bioactivity, excellent cytocompatibility	[76]
ALG, Agarose	E-beam; acceleration energy: 10 MeV; pulse repetition rate: 180 Hz; doses: 10, 20, and 30 kGy	TE	Good mechanical and dynamic properties	[77]
Porcine corneal tissues	E-beam; acceleration energy: 2.6 MeV; dose: 25 kGy; dose rate: 5 kGy/pass	TE	Improved structural and biomechanical properties, high biocompatibility	[78]
AAm, acrylonitrile, PP	E-beam; acceleration energy: 2 MeV; doses: 40, 60, 80, 100, 160, and 200 kGy; dose rate: 200 Gy/s	Biomedicine, cell harvesting	Thermo-responsive swelling, non-cytotoxicity	[79]
COL, PVP, PEO, CMC, CHT	E-beam; acceleration energy: 6 MeV; beam current: 5 μA; pulse repetition: 53 Hz; doses: 5, 10, 20, 30, and 40 kGy; dose rate: 4 kGy/min	TE, wound dressing	Excellent stability, elastic structure, adequate swelling properties, excellent biocompatibility	[80]
TG, PVA, HNT	E-beam; acceleration energy: 10 MeV; dose: 28 kGy	Bone tissue engineering	Porous microstructure, adequate degradation rate, superior mechanical properties, excellent cell viability	[81]
PMeOx, EnOx	E-beam; acceleration energy: 10 MeV; pulse repetition: 423 Hz; doses: 2, 5, 10, 20, 40, and 100 kGy; dose rate: 0.5 kGy/min	Living tissue	Excellent non-biofouling properties, adequate mechanical behavior, good stability	[82]
CS, PVP, PEO, PAA, AgNP	E-beam; acceleration energy: 6 MeV; dose: 15 kGy; dose rate: 3 kGy/min	TE, wound dressing	Significant antimicrobial activity, high biocompatibility, excellent stability and swelling capacity	[83]

**Table 2:** Raw materials, fabrication technologies, applications, and outcomes of various studies utilizing γ-irradiation.

Materials	Fabrication Method	Applications	Findings	References
P-ECM; enzyme	γ-sterilization; radiation doses: 5 + 2 kGy, 15 + 2 kGy, and 25 + 2 kGy	Soft tissue reconstruction	Biocompatibility, physicochemical characteristics	[86]
SF; dECM	γ-irradiation; dose: 60 kGy; dose rate: 196 Gy/min; room temperature	Cartilage regeneration	Higher water absorption capacity, surface wettability, and no cytotoxicity	[90]
Zein; PVB	γ-irradiation-induced copolymerization; doses: up to 50 kGy; dose rate: 1.33 kGy/h	TE	Thermal stability, Vero (monkey kidney cell) proliferation	[91]
PVP; CMC	γ-irradiation; doses: 25 kGy and 40 kGy; dose rate: 5–6 kGy/h	Antimicrobial burn wound dressing	Antimicrobial efficacy, fluid absorption ability, moisture transmission ability	[92]
GO; PVP; PCL	γ-irradiation; dose: 25 kGy/h	Promising for bone TE	Excellent mineralization, BMP-2 release, osteodifferentiation	[93]
pHEMA; AA; Fe^3+^	γ-sterilization; dose: 25 kGy	Biomedical applications or TE	Good wound healing, no distinct inflammatory properties	[94]
Aramid or PBO; PVA; Mg-substituted ceramic (β-TCP or BCP)	γ-sterilization; dose: 25 kGy; dose rate: 5 kGy/h	Bilayer composites for the replacement of osteochondral defects	High biomimicry with natural tissues, biological behavior, adequate porosity	[88]
NC; ALG; HA	Autoclave, β-radiation, and γ-radiation; beam energy: 10 MeV; maximum power: 80 kW; dose: 25 kGy; dose rate: 3.2 Gy·min^−1^	3D bioprinted scaffolds for TE and regenerative medicine	Sterilization-induced biocompatibility and higher cell viability against D1-MSCs	[95]
ECM	Seeding of hMVECs into the 3D blood vessel model for 4 days before and after γ-irradiation; doses: 4 Gy and 8 Gy	Microfluidic chips for evaluation of γ-irradiation on angiogenic and endothelial barrier properties	Reorganization of the fiber structure of hMVECs, increase in apoptotic hMVECs	[87]
GEL	γ-sterilization; dose: 25 kGy; sterilization time: 3790 min	Potential for TE	Swelling, compressive modulus	[96]
SF	γ-radiation; doses: 45, 75, 105, 135, and 165 kGy; dose rate: 196 Gy/min; room temperature	Soft/tough tissue engineering scaffold	Tunable porosity, uniform pore structure, adjustable mechanical strength, good biocompatibility	[97]
Gel; PEG; HEC; CHT	γ-sterilization; dose: 25 kGy	Potential for TE	Water uptake, mechanical properties, stress relaxation response	[89]
Mg-doped Hap; AG; GEL	γ-irradiation source	Bone tissue regeneration	Biocompatibility, antibacterial activity, in vitro drug release	[98]
AG; TG; gentamicin	γ-irradiation; dose: 27.3 kGy	Wound dressing potential	Mucoadhesive, antioxidant/antibacterial activity	[99]
Copoly (NIPAAm/DMAAm/MMA), copoly (NIPAAm/DMAAm/EEM)	γ-rays; dose: 50 kGy; dose rate: 10.1 kGy/h; room temperature	Biomedical sensor application	Thermo-responsive hydrogel, swelling characteristics	[100]
RGD-functionalized γ-irradiated ALG	γ-irradiation; dose: 5 Mrad	Osteochondral defect regeneration	Resistance to hypertrophy, vascularization, and endochondral ossification	[101]
Cellulose; PVP; PEG	γ-irradiation; dose: 50 kGy; dose rate: 3.6 kGy/h	Connective tissue regeneration applications; terpolymer scaffolds for cell attachment	Higher cell viability of fibroblasts at low concentrations of PVP/PEG	[102]
GEL; CHT; HA	Freeze-drying; γ- irradiation sterilization; dose: 25 kGy	Hybrid biomaterial for TE: scaffold with autologous hydrogel carrying hMSCs	Partial biodegradation at one week, high biocompatibility, early regeneration capacity at 4 weeks, absence of rejection signs	[103]
SF; HAP	γ-ray irradiation; dose: 60 kGy; dose rate: 15 kGy/h	Bone TE	Increased osteogenic differentiation, improved cell adhesion and proliferation	[104]
RGD-functionalized γ-irradiated ALG PEGMA; GelMA; PCL	γ-ray irradiation; dose: 5 Mrad 3D bioprinting	3D bioprinting; whole bone organ engineering	Mechanically reinforced hypertrophic cartilage templates	[105]
PEG; CHT	γ-sterilization; dose: 13.83 ± 0.7 kGy	TE application	Biocompatibility, hemocompatibility	[106]
HA; CS; PAAc	γ-irradiation; doses: 5–25 kGy; nitrogen atmosphere; room temperature	Skin TE	Gel fractions, high water content, HaCaT cell viability	[107]
COL	γ-irradiation; dose rate: 20 Gy/min; room temperature	Potential for TE applications	Accelerated apoptosis or L929 cell proliferation	[108]
HA; CS; PVA	γ-irradiation; doses: 5–25 kGy; nitrogen atmosphere; room temperature	Skin TE	High cell viability, cell growth after 7 days	[109]
COL; DEX	γ-irradiation; dose rate: 15 Gy/min; room temperature	Artificial dermal substitutes	Tunable mechanical strength, pore size, water absorption, controllable degradation rate	[110]
PLCL; GEL; AAc	γ-irradiation; dose: 10 kGy; ambient temperatures; electrospinning	Vascular TE	Significant increase in Young’s modulus, improved SMC infiltration and proliferation	[111]
nHA; GEL; AAc	Copolymerization and crosslinking through γ-irradiation; dose rate: 10.28 kGy/h	Bone TE scaffolds	In situ nanocomposite formation, biocompatibility	[112]
ALG	γ-irradiation; doses: 0–30 kGy	Potential for wound dressing	Higher flexibility, better pore structure control, hydration properties, and mechanical integrity compared to foams prepared using other techniques	[113]
PGA; apatite	γ-ray irradiation; doses: 40, 80, and 120 kGy; room temperature; under nitrogen	TE applications	Good cell proliferation of MC3T3-E1 cells after 7 days	[114]
Hydroxypropyl cellulose	γ-irradiation; dose rate: 10 kGy/h; irradiation time: 30 min	Soft TE	High water content, interconnected macroporosity, mechanical integrity, and cytocompatibility	[115]
PEG	γ-sterilization	Potential for TE and controlled drug delivery	Release of cyclosporine, rhodamine B	[34]
GMA-DEX; GEL; BMP	γ-ray irradiation; dose: 1 kGy	Potential for TE and drug delivery applications	Temperature responsiveness, BMP release	[116]

## Data Availability

Not applicable.
